# Reading the mind of children in response to food advertising: a cross-sectional study of Malaysian schoolchildren’s attitudes towards food and beverages advertising on television

**DOI:** 10.1186/s12889-015-2392-z

**Published:** 2015-10-12

**Authors:** See Hoe Ng, Bridget Kelly, Chee Hee Se, Sharmela Sahathevan, Karuthan Chinna, Mohd Noor Ismail, Tilakavati Karupaiah

**Affiliations:** Dietetics Programme, School of Healthcare Sciences, Faculty of Health Sciences, National University of Malaysia, Kuala Lumpur, Malaysia; Early Start Research Institute, Faculty of Social Sciences, University of Wollongong, Wollongong, NSW Australia; Julius Centre University Malaya, Department of Social and Preventive Medicine, University of Malaya, Kuala Lumpur, Malaysia; School of Hospitality, Tourism and Culinary Arts, Taylor’s University, Selangor Darul Ehsan, Malaysia

**Keywords:** Television food advertising, Advertisement recognition, Favourite advertisement, Purchase request, Product preference, Obesogenic environment, Appealing food advertisement

## Abstract

**Background:**

Television food advertising (TVFA) is the most dominant medium in the obesogenic environment promoting unhealthy food choices in children.

**Methods:**

This cross-sectional study investigated children’s attitudes towards TVFA by examining four well-cited induction factors namely advertisement recognition, favourite advertisement, purchase request, and product preference. Malaysian urban schoolchildren (7 to 12 years) of equal ethnic distribution were voluntarily recruited (*n* = 402). Questionnaire administration was facilitated using a food album of 24 advertised food products.

**Results:**

Majority of children were older (66.2 %), girls (56.7 %) with one-third either overweight or obese. TV viewing time for weekend was greater than weekdays (4.77 ± 2.60 *vs* 2.35 ± 1.40 h/day) and Malay children spent more time watching TV compared to Chinese (*p* < 0.001) and Indian (*p* < 0.05) children. Chinese children spent significantly more time surfing the internet compared to either Malay or Indian (*p* < 0.01). Median score trend was advertisement recognition > favourite advertisement and product preference > purchase request, and significantly greater (*p* < 0.001) for non-core than core food advertisements. TV viewing time and ethnicity significantly influenced all induction factors for non-core foods. After correcting for all influencing factors, ‘favourite advertisement’ (IRR_final adj_: 1.06; 95 % CI: 1.04 to 1.08), ‘purchase request’ (IRR_final adj_: 1.06; 95 % CI: 1.04 to 1.08) and ‘product preference’ (IRR_final adj_: 1.04; 95 % CI: 1.02 to 1.07) still were significantly associated with TV viewing time. For every additional hour of TV viewing, the incidence rates increased significantly by 1.04 to 1.06 for ‘favourite advertisement’, ‘purchase request’ and ‘product preference’ related to non-core foods amongst Malay and Indian children. However, Chinese children only demonstrated a significant association between TV viewing time and ‘favourite advertisement’ (IRR_adj_: 1.06; 95 % CI: 1.01 to 1.10).

**Conclusion:**

This study highlights TVFA as a powerful medium predisposing the mind of children to non-core foods through appealing TV commercials, promoting purchase request and generating unhealthy food preferences in early childhood.

**Electronic supplementary material:**

The online version of this article (doi:10.1186/s12889-015-2392-z) contains supplementary material, which is available to authorized users.

## Background

The global burden of non-communicable disease (NCDs) is a deep concern to the public health community especially when driven by escalating rates for overweight and obesity in children, occurring across many regions and nations as reported by a systematic analysis of obesity prevalence data from 1980 to 2013 [1]. Malaysia has the highest rates of obesity in South East Asia, with estimated prevalence rates of 22.5 % in boys and 19.1 % in girls who are less than 20 years old. This is a serious problem as NCDs such as diabetes, hypertension, fatty liver, and cardiovascular diseases of adulthood are associated with childhood obesity [2, 3]. The obesogenic environment of childhood in this context deserves attention as a modifiable risk factor. It is a combination of influences that promote obesity from all surroundings, opportunities or conditions of life [4]. Determinants of obesity are broad, and include cultural, environmental and psychological triggers, as described in the UK Foresight Report [5]. Within this matrix of determinants, the social psychology cluster inclusive of television (TV) watching and media availability is influential at the societal level. In this environment, it is well understood that TV food advertising (TVFA) is the most dominant medium to promote unhealthy foods and food choices to young viewers [6–8].

Over the past three decades, marketing has evolved considerably in the food environment to accommodate the food industry’s definition on what is acceptable and desirable to eat [9]. Generally, food advertising portrays food advertisements as exciting and fun, and users of these products as equally appealing [10]. A systematic review on the nature, extent and effects of food marketing to children has indicated that most food advertising directed to children are those low in positive nutrients and energy-dense [11]. Techniques such as animation, story-telling, visual effects, premium offers (e.g. free toys) and product endorsements with licensed characters or movie industry tie-ins have been shown to influence children [10, 12]. It is thus observed that animation used in marketing aims to capture the attention of children by merging the fictional world of advertisements and the real world. Advertising on TV also penetrates the mind of children more easily than other static media, as they are more likely to develop a receptive memory with visual simulation generated by TV [13].

Evidence from scientific literature suggests that food marketing influences children in various ways. Qualitative surveys on children’s natural reaction when exposed to TVFA has revealed modest evidence that advertising attracts children’s attention and enhances acceptance, preference and demand for advertised products [11]. Additionally, an experimental study concluded TVFA increased children’s food consumption when they watched food advertisements and with a greater likelihood for overweight and obese children to consume energy-dense snacks compared to normal weight children [14]. Of concern, the lasting effect of early TVFA exposure leads to the development of unhealthy eating habits, mediated by perceived taste for highly advertised unhealthy food [15]. The aetiology of this pattern would be through increasing product awareness, generating positive attitudes towards junk foods, influencing children’s food preferences, and arousing cues for purchasing requests as noted in some studies [7, 16–18].

Children’s age is a major factor in their ability to comprehend TVFA messages. Children were found capable of recognising TV commercials as early as six years old, through identifying short breaks between programmes using cues such as voice-over, jingles, pace, and editing [19, 20]. Food advertising messages may then also be reinforced through watching TV advertisements, as evidenced by parents’ observations that their children repeat an advertised product’s slogans or taglines during their daily lives [13]. It is hypothesised that children need to acquire three ascending levels of understanding before they are truly able to comprehend messages portrayed by food advertisers [21]. These include: (i) the ability to distinguish programme content from commercials; (ii) the ability to recognise the basic intention of food advertising, which is to sell or promote their products (to distinguish selling intent) and; (iii) scepticism and awareness of biased messages related to advertised food products (to distinguish persuasive intent). Children up to 12 years old are still vulnerable to food advertising and less able to be sceptical about any message content [22]. Recent scientific evidence emphasise restriction of food promotion targeting children up to 12 years of age [21, 23].

The effectiveness of TVFA directed to children can be attributed to several influencing factors. Amongst these, it is suggested that the impact of advertising message on children may differ due to sociocultural differences, but there is a gap in knowledge regarding this aspect [24]. In California, an ethnic-specific content analysis of TV channels indicated Spanish-language TV channels were dominated by fast foods (~30 %), followed by breakfast cereals and candy [25]. According to Kent et al. [26], French-speaking children in Quebec, Canada were exposed to lesser high fat, sugar, or sodium (81.0 %) food advertisements compared to English-speaking children from Ontario (89.8 %) and Quebec (96.6 %). This phenomenon warrants a need to measure the influence of food marketing based on ethnicity related to minority populations and this should be taken into account in policy development [27]. A study reported that children of Muslim cultures in the Middle East are governed by their parents in relation to moderating purchasing requests [13], whereas in Western countries ‘pestering’ behaviours are noted [7]. Gender is another influencing factor as girls are shown to be more resistant to advertising messages and persuasion compared to boys, as shown for fast food advertising [8]. The availability of TV sets within children’s bedrooms is linked to higher screen time [28], which predicts future requests for advertised food products [29]. Lastly, children’s pocket money would be another influencing factor related to the purchasing power of children for unhealthy food items [30].

Observational studies indicate that Malaysian TV food marketing is dominated by foods high in fat, refined sugars, and salt (HFSS), such as sugar sweetened beverages, unhealthy snacks, confectionery, instant noodles, biscuits and chocolate [31, 32]. These are unhealthy foods and designated as ‘non-core’ as opposed to ‘core’ foods which are healthy foods [33]. However, the impact of this type of TVFA on Malaysian children has not been studied extensively. Further, a recent multi-country study comparing TVFA patterns across Asian countries substantiated that non-core foods dominated TVFA in Malaysia, with a ratio of 7.6 non-core food advertisements for every one advertisement for core foods [34]. Given the gap in knowledge relating to the Malaysian obesogenic environment, there is a strong justification to investigate the impact of TVFA on children in Malaysia. This study aimed to provide a snapshot of children’s attitudes about TVFA of non-core foods by evaluating four induction factors and the relationship of these factors with TV viewing duration. The four induction factors include (i) recognition of advertised food products (advertisement recognition), (ii) liking the food advertisements on TV (favourite advertisement), (iii) purchase requests induced by TVFA (purchase request), and (iv) product preference generated by TVFA (product preference). These factors were drawn from a literature review [7, 16–18] and we used the term ‘induction’ as an appropriate label for these factors. In contrast, this study also explores the relationships between potential influencing factors such as gender, age, ethnicity, body mass index and the cited four induction factors.

## Methods

### Study design, ethics statement, and subject recruitment

According to the census conducted in 2010, Federal Territory of Kuala Lumpur was reported to have a population of 1,517,998 and it was the highest population density (6891 persons per square km) in Malaysia [35]. Additionally, childhood obesity in Kuala Lumpur is well-studied and a major public health concern was that about 34 % children were overweight or obese [36, 37]. For these reasons, it was decided that a cross-sectional study would be conducted in Kuala Lumpur. Ethical approval was obtained from the Research Ethics Committee of National University of Malaysia (UKM NN-070-2013) and approval to conduct the study in government-run schools was sought from the Ministry of Education and school administrators.

**Fig. 1:**
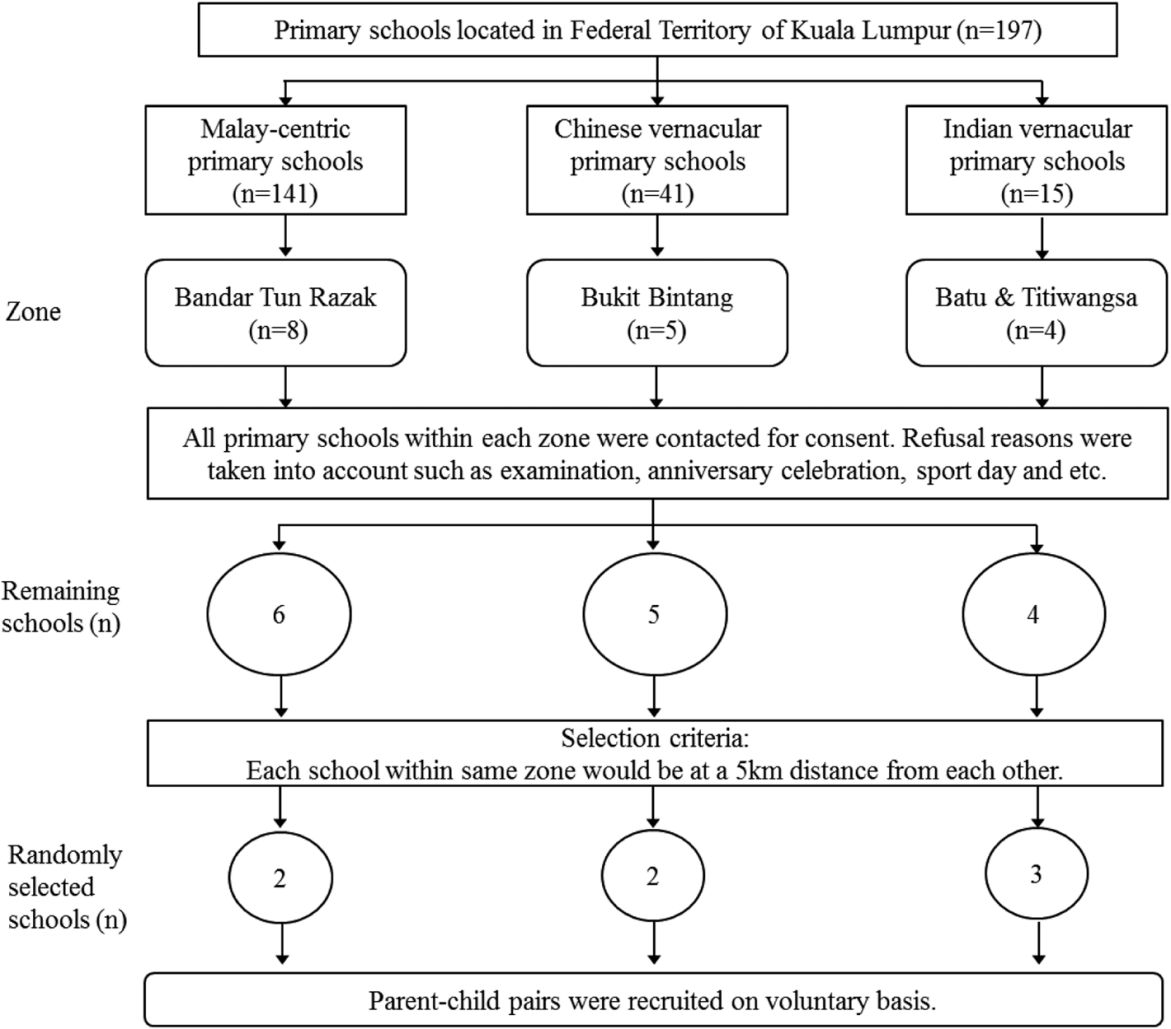
The flowchart of school selection. Abbreviation: *n* = number of schools

First, the list of primary schools registered with the Ministry of Education in Federal Territory of Kuala Lumpur was obtained. As per the source, there were 197 primary schools in this location, of which 141 schools were national, Malay-centric primary schools, followed by 41 Chinese and 15 Indian vernacular schools [38]. In order to better capture the impact of TVFA on children, Gregori et al. [39] stressed that understanding the influence of multi-cultures and relation to patterns of eating should be a fundamental objective of research design. Hence, in this study, the schools were clustered into 3 zones as per demographic ethnic group based on locality: there were eight schools in Bandar Tun Razak (mainly Malays), five in Bukit Bintang (mainly Chinese), and four in Batu and Titiwangsa (mainly Indians). Within each cluster, all the school headmasters or headmistresses were contacted for consent. Of the 17 schools, after taking account of refusal reasons such as interfering with school activities, sport days, and examination period, 15 schools consented to participate in the study. Within each cluster, schools were selected randomly: two from Bandar Tun Razak, two from Bukit Bintang, and three from Batu and Titiwangsa. Data collection was carried out from June to December 2013. Figure 1 shows the flowchart of school selection for this study.

### Questionnaire development

An assessment on the impact of food advertising related to children’s food preference is suggested to be more comprehensive and realistic with a questionnaire-based tool [14]. We therefore designed an interviewer-administered questionnaire to assess the impact of TVFA related to children’s food preferences. The initial version of the questionnaire was drafted in English content and face validity of the questionnaire were established through parents, teachers and nutrition experts. The final version of the questionnaire was translated into Malay and Mandarin languages by two research dietitians, who were native speakers of these two languages. The modified questionnaire was subsequently pilot-tested for clarity amongst 30 children from the different ethnic denominations. A copy of this final questionnaire is available as an additional file (Additional file 1).

The questionnaire was organised to provide:

(i) *Background information-* Demographic details such as gender, ethnic, age and school details were included. Children were asked about their frequency and type(s) of physical activity carried out in the past one week. Time spent on physical activity and internet surfing was recorded. A questionnaire sheet was included mainly to probe for the amount of children’s TV viewing time. In the sheet, one blank clock figure for a.m. period and one blank clock figure for p.m. period were designated separately for weekdays and weekend. As indicated by a sample clock, time was quantified by the child for each TV viewing session by a ‘triangulation’ of starting and completion times with the centre of the clock. More than one triangle could be drawn per clock to designate different TV viewing sessions. Time was quantified for each TV viewing session by subtracting the starting time from the completion time of viewing. The amount of TV viewing time was quantified in hours.
(ii) *Induction factors and food advertisement album –* In order to probe induction factors, we utilised a food album constructed of most frequently advertised core and non-core food products. Core foods are defined as low-calorie, nutrient-dense food products, while non-core foods are foods high in fat, refined sugars, and salt (HFSS) [33]. Table 1 lists the food products included in the food album, which comprised 24 food products including 9 core or healthy foods and 15 non-core or unhealthy foods. These food products were selected based on their advertising frequency as determined through a content analysis of TVFA data on Malaysian free-to-air channels collected between September and October 2012 [34]. The disproportionate ratio between core to non-core food products in the food album was reflective of the dominating non-core food advertising on Malaysian TV channels. Subjects were required to indicate ‘yes’ (coded as “1”) or ‘no’ (coded as “0”) for each listed food product on the questionnaire sheet by referring to the food album for pictures of the advertised product. This feedback was obtained for each induction factor which included (i) advertisement recognition (ii) favourite advertisement (iii) purchase request, and (iv) product preference.

**Table 1:** Food advertisements listing in the food album

Core food products (*n* = 9)		Non-core food products (*n* = 15)	
FP1	Low sugar, high fibre breakfast cereal (Brand 1)	FP2	Cultured milk food with added sugar
FP6	Plain bread (Brand 1)	FP3	High sugar breakfast cereal
FP9	Plant based margarine	FP4	Chocolate
FP13	Frozen low fat yoghurt	FP5	Cultured drink with high sugar content
FP14	Low sugar, high fibre breakfast cereal (Brand 2)	FP7	Sugar-sweetened soft drink
FP17	Plain bread (Brand 2)	FP8	Processed crispy fried chicken
FP18	Plain cream cracker	FP10	Sweet biscuit
FP21	Soya bean milk	FP11	Sweet cake
FP22	Rice, plain	FP12	Extruded snack (Brand 1)
		FP15	Ice-confectionery
		FP16	Ice-cream
		FP19	Sugar-sweetened beverage
		FP20	Extruded snack (Brand 2)
		FP23	Fast food
		FP24	Instant noodle

*FP* = food product (range 1–24 as per randomised sequence of food products shown in the food album)

Note: Core foods are defined as low-calorie, nutrient-dense food products, while non-core foods are foods high in fat, refined sugars, and salt (HFSS) [33]

### Interviewing protocol

A signed written consent form was obtained from parents or guardians prior to proceeding with the data collection on children. Caregivers were required to provide sociodemographic information on monthly household income and pocket money given to their child daily. Children were selected if they met the following criteria: (i) watched TV at least half an hour per week, (ii) were physically and mentally healthy and (iii) only one child per household could participate. Carter et al. [23] suggest a small group interview facilitates a better exploration of children’s thoughts as this helps them to be at ease and help each other out if required. This approach was adopted in the study design by which subjects were randomly assigned into small groups of four to six boys and girls of similar age in each session. Each group session was facilitated by at least two trained interviewers. However, subjects were informed not to reveal their responses to others in the group but provide written answers in the questionnaire to prevent peer bias.

The role of trained facilitators was to probe through questions emphasising the impact of TVFA on each induction factor for each food product shown in the food album. For example, for (i) advertisement recognition – “*Have you seen this TV advert before?*”; (ii) favourite advertisement – “*Do you like the advert?*”; (iii) purchase request – “*Will you ask your parent to buy it?*”; and (iv) product preference – “*Do you like to eat or drink this food product?*”. Subjects were also asked to choose reason(s) from a list of ten as to why they liked advertised food products shown. The list included *tasty, good for health, cartoon (endorsed by promotional character), free gifts (premium offered), storyline, music, jingles (slogan or catchy songs) or colourful visuals used during food advertisements, special effects (such as animation*). Any reason not within this list was to be listed under *others*.

### Anthropometric measurements

Weight measurements using an electronic TANITA HD-309 digital scale (TANITA Corporation, Japan) were recorded to the nearest 0.5 kg, whilst measurements for height using a SECA 206 body-meter (Seca GmbH & Co. KG., Germany) were recorded to the nearest 0.1 cm. Data for weight and height measurements were transferred into AnthroPlus software (World Health Organization) to compute body mass index (BMI). Z-scores for BMI were computed with this software which uses cut-points specific to gender and age as per the World Health Organization [40].

### Data interpretation

All time-related factors such as physical activity, internet surfing and TV viewing during the past one week were reported as daily time spent in hours. Subject response to each induction factor was differentiated as per core (*n* = 9) and non-core (*n* = 15) food categories. Median scores for each induction factor (ranging from 0.00 to 1.00) were computed based on sum of food items in agreement (*yes*) divided by total number of food products for core or non-core food categories.

### Statistical analysis

Analyses were conducted using Statistical Package for Social Sciences, version 16.0 (SPSS Statistics Inc. Chicago IL. USA). Demographic variables such as ethnic, age, gender, BMI, TV viewing duration, TV set in the bedroom, internet surfing time, physical activity, daily pocket money, and monthly household income were described as percentage (%) or mean ± SD. Ethnic differences in time spent on TV viewing, physical activity and internet surfing were analysed using one-way ANOVA and Kruskal Wallis Test. When significant, *post-hoc* analyses were performed using Tukey (if Levene test *p* > 0.05) or Dunnett T3 (if Levene test *p* < 0.05) to identify the pairwise difference. Wilcoxon signed-rank test was used to determine the median score differences between core and non-core foods for each induction factor.

The four induction factors considered in this study are (i) advertisement recognition, (ii) favourite advertisement, (iii) purchase request, and (iv) product preference, with each of these factors as a count measure. To identify factors influencing the induction factors, Poisson regression was used. If the assumption for Poisson regression was not met, the negative binomial regression procedure was applied. For ease interpretation, incidence rate ratios (IRR) were reported. The influencing factors considered were TV viewing time, age, gender, ethnicity, TV set in bedroom, physical activity, internet surfing time, daily pocket money of child, and monthly household income. Analyses were also done to test the association between TV viewing time and the induction factors, controlling for all other demographic variables. The effect of TV viewing time on the induction factors were also tested for each ethnic group, separately. A *p*-value of 0.05 was considered to be statistically significant.

## Results

### Children’s characteristics

A total of 402 primary schoolchildren participated in the survey (Table 2). The majority of subjects were girls (56.7 %) and were equally distributed amongst the three ethnic groups (Malay = 38.3 %, Chinese = 31.1 % and Indian = 30.6 %). The average age was 9.85 ± 1.38 years and nearly two-thirds of children were more than 9 years old. More than half the children (54.5 %) had a normal BMI, whilst one-third were classified as possible risk of overweight (15.7 %) and overweight or obese (18.9 %). Children’s pocket money averaged RM 3.13 ± 1.80 (approximately USD 1.00 ± 0.60) daily.

**Table 2:** Demographic data of children (*n* = 402)

Characteristics	*n* (%)	Mean ± S.D.
Gender		
Boys	174 (43.3)	-
Girls	228 (56.7)	-
Ethnic		
Malay	154 (38.3)	-
Chinese	125 (31.1)	-
Indian	123 (30.6)	-
Age (year)	402 (100.0)	9.85 ± 1.38
Younger children (≤9 years old)	136 (33.8)	8.22 ± 0.84
Older children (>9 years old)	266 (66.2)	10.7 ± 0.69
Body Mass Index, BMI^a^		
Severely wasted and wasted (Below −2 Z-score)	44 (10.9)	-
Normal (1 ≤ Z-score ≤ −2)	219 (54.5)	-
Possible risk of overweight (1 < Z-score ≤ 2)	63 (15.7)	-
Overweight and obese (Above 2 Z-score)	76 (18.9)	-
Daily Physical Activity (hrs)	402 (100.0)	1.15 ± 0.68
Less than once weekly	24 (6.0)	0.82 ± 0.50
1–3 times weekly	245 (60.9)	1.03 ± 0.58
4–6 times weekly	78 (19.4)	1.30 ± 0.72
Everyday	55 (13.7)	1.58 ± 0.83
Daily Internet Surfing^b^ (hrs)	230 (100.0)	1.65 ± 1.58
Daily TV Viewing time (hrs)	402 (100.0)	3.03 ± 1.52
Weekday (hrs)	402 (100.0)	2.35 ± 1.40
Weekend (hrs)	402 (100.0)	4.77 ± 2.60
Television in Bedroom		
Yes	84 (20.9)	-
No	318 (79.1)	-
Daily Pocket Money (RM)	402 (100.0)	3.13 ± 1.80

^a^Categories were based on Z-score of WHO classification [40]. Two new categories were formed due to very few subjects (severely wasted merged with wasted category; overweight merged with obese category)

^b^Only 260 subjects reported having internet access at home, but only 230 children were allowed online access by parents/ guardians

**Fig. 2:**
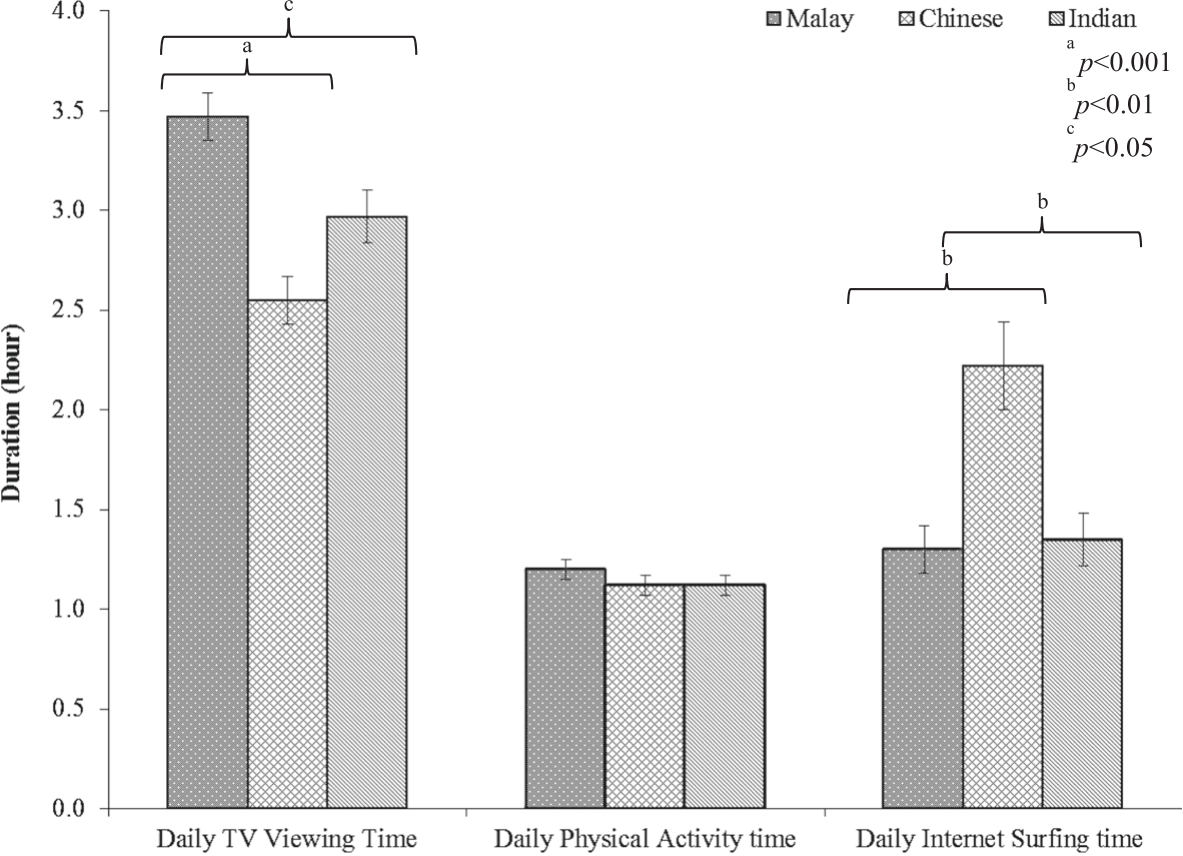
Distribution of time spent daily by children. The average time spent daily (hour ± standard error of mean) by children as per ethnic groups for TV viewing, physical activity and internet surfing (only analysed children who reported using internet, *n* = 230)

Subjects spent an average 1.15 ± 0.68 h performing physical activity daily. Additionally, 230 subjects reported spending an average of 1.65 ± 1.58 h on the internet each day. TV viewing was more intense during weekend (4.77 ± 2.60 h) compared to weekdays (2.35 ± 1.40 h) with an average TV viewing time of 3.03 ± 1.52 h daily. In addition, one-fifth of subjects (20.9 %) reported having a TV in their bedroom. Figure 2 shows Malay children spent more time watching TV compared to Chinese (*p* < 0.001) and Indian (*p* < 0.05) children. In contrast, Chinese children spent significantly more time surfing internet compared to Malay and Indian children (*p* < 0.01).

### Induction factors shaping attitudes towards advertised food categories

Table 3 indicates median scores (25^th^ percentile to 75^th^ percentile) for all induction factors as per core and non-core food categories. Notably, a total of 191 children (47.5 %) reported spending three hours or more watching TV daily. Overall, advertisement recognition achieved the highest scores among induction factors as subjects significantly recognised more non-core foods compared to core foods (*p* < 0.001) as evidenced by a score of 0.93 (0.80–1.00) for non-core foods compared to 0.78 (0.56–0.89) for core foods. For the induction factor ‘favourite advertisement’, a similar pattern was observed with the median score for non-core foods (0.73, 0.53–0.87) remaining significantly greater (*p* < 0.001) than for the core foods category (0.44, 0.22–0.56). This trend was also repeated for ‘purchase request’ (non-core foods = 0.60 (0.40–0.80) > core foods = 0.33 (0.11–0.44); *p* < 0.001) and for ‘product preference’ (non-core foods = 0.73 (0.60–0.87) > core foods = 0.44 (0.22–0.56); *p* < 0.001). Amongst all induction factors the lowest median score was for core foods ‘purchase request (0.33, 0.11–0.44). Notably, scores for non-core compared to core foods for all induction factors were significantly higher (*p* < 0.001) for all sub-groups by TV viewing time, gender, ethnic, age, children’s BMI, TV set in the bedroom, daily physical activity, daily pocket money, daily internet surfing time and monthly household income. Therefore, the following analyses focuses only on examining induction factors related to non-core food advertising.

**Table 3:** Proportion of induction factors’ score as per food advertisement categories

Influencing factors	Advertisement Recognition		Favourite Advertisement		Purchase Request		Product Preference	
	Core	Non-core	Core	Non-core	Core	Non-core	Core	Non-core
	M (IQR)	M (IQR)	M (IQR)	M (IQR)	M (IQR)	M (IQR)	M (IQR)	M (IQR)
Overall	0.78	0.93	0.44	0.73	0.33	0.60	0.44	0.73
	(0.56–0.89)	(0.80–1.00)	(0.22–0.56)	(0.53–0.87)	(0.11–0.44)	(0.40–0.80)	(0.22–0.56)	(0.60–0.87)
Duration of TV viewing^a^								
<3 h (*n* = 211)	0.67	0.93	0.33	0.67	0.33	0.60	0.33	0.67
	(0.56–0.89)	(0.80–1.00)	(0.22–0.56)	(0.47–0.80)	(0.11–0.44)	(0.40–0.73)	(0.22–0.56)	(0.53–0.80)
≥3 h (*n* = 191)	0.78	1.00	0.44	0.80	0.33	0.67	0.44	0.80
	(0.56–0.89)	(0.87–1.00)	(0.33–0.67)	(0.60–0.93)	(0.22–0.44)	(0.47–0.80)	(0.33–0.56)	(0.67–0.87)
Gender								
Boys (*n* = 174)	0.67	0.93	0.33	0.73	0.33	0.63	0.44	0.73
	(0.44–0.89)	(0.85–1.00)	(0.22–0.56)	(0.58–0.87)	(0.19–0.44)	(0.40–0.80)	(0.22–0.56)	(0.60–0.87)
Girls (*n* = 228)	0.78	0.93	0.44	0.73	0.33	0.60	0.44	0.67
	(0.56–0.89)	(0.80–1.00)	(0.22–0.56)	(0.53–0.87)	(0.11–0.44)	(0.40–0.73)	(0.22–0.55)	(0.55–0.80)
Ethnic								
Malay (*n* = 154)	0.89	1.00	0.44	0.80	0.33	0.67	0.44	0.80
	(0.67–1.00)	(0.93–1.00)	(0.33–0.67)	(0.60–0.93)	(0.22–0.56)	(0.47–0.80)	(0.33–0.67)	(0.60–0.93)
Chinese (*n* = 125)	0.67	0.93	0.33	0.67	0.22	0.53	0.33	0.67
	(0.56–0.89)	(0.80–1.00)	(0.11–0.44)	(0.47–0.80)	(0.11–0.44)	(0.36–0.70)	(0.22–0.44)	(0.53–0.80)
Indian (*n* = 123)	0.56	0.80	0.44	0.73	0.22	0.60	0.33	0.67
	(0.33–0.78)	(0.73–1.00)	(0.22–0.56)	(0.60–0.87)	(0.11–0.44)	(0.40–0.73)	(0.22–0.56)	(0.60–0.80)
Age								
Younger children (*n* = 136)	0.67	0.93	0.44	0.73	0.33	0.67	0.44	0.73
	(0.47–0.89)	(0.80–1.00)	(0.22–0.67)	(0.60–0.93)	(0.22–0.56)	(0.47–0.80)	(0.25–0.67)	(0.60–0.87)
Older children (*n* = 266)	0.78	0.93	0.33	0.73	0.33	0.60	0.44	0.73
	(0.56–0.89)	(0.80–1.00)	(0.22–0.56)	(0.53–0.87)	(0.11–0.44)	(0.40–0.73)	(0.22–0.56)	(0.58–0.87)
Body Mass Index, BMI								
Severely wasted and wasted (*n* = 44)	0.78	0.93	0.33	0.73	0.33	0.53	0.33	0.67
	(0.56–0.89)	(0.87–1.00)	(0.25–0.56)	(0.53–0.80)	(0.22–0.44)	(0.40–0.73)	(0.33–0.56)	(0.55–0.85)
Normal (*n* = 219)	0.78	0.93	0.44	0.73	0.33	0.60	0.44	0.73
	(0.56–0.89)	(0.87–1.00)	(0.22–0.67)	(0.53–0.93)	(0.11–0.44)	(0.47–0.80)	(0.22–0.56)	(0.60–0.87)
Possible risk of overweight (*n* = 63)	0.78	0.93	0.33	0.73	0.22	0.53	0.44	0.80
	(0.56–0.89)	(0.80–1.00)	(0.22–0.56)	(0.53–0.87)	(0.11–0.33)	(0.40–0.80)	(0.22–0.56)	(0.60–0.87)
Overweight and obese (*n* = 76)	0.72	0.93	0.33	0.73	0.33	0.60	0.44	0.70
	(0.44–0.89)	(0.75–1.00)	(0.22–0.56)	(0.53–0.80)	(0.22–0.53)	(0.40–0.73)	(0.22–0.56)	(0.53–0.80)
TV Set in Bedroom								
No TV in bedroom (*n* = 318)	0.78	0.93	0.44	0.73	0.33	0.60	0.44	0.73
	(0.56–0.89)	(0.80–1.00)	(0.22–0.56)	(0.53–0.87)	(0.11–0.44)	(0.40–0.80)	(0.22–0.56)	(0.60–0.87)
TV in bedroom (*n* = 84)	0.78	1.00	0.44	0.73	0.33	0.63	0.39	0.39
	(0.56–0.89)	(0.87–1.00)	(0.22–0.56)	(0.60–0.87)	(0.22–0.44)	(0.40–0.80)	(0.22–0.56)	(0.53–0.85)
Physical Activity Level (PAL)^a^								
PAL ≤1 h (*n* = 246)	0.67	0.93	0.39	0.73	0.33	0.60	0.44	0.73
	(0.44–0.89)	(0.87–1.00)	(0.22–0.56)	(0.53–0.87)	(0.11–0.44)	(0.40–0.80)	(0.22–0.56)	(0.60–0.87)
PAL >1 h (*n* = 156)	0.78	0.93	0.44	0.70	0.33	0.60	0.44	0.73
	(0.56–0.89)	(0.87–1.00)	(0.22–0.56)	(0.53–0.87)	(0.22–0.44)	(0.40–0.80)	(0.33–0.56)	(0.55–0.87)
Pocket Money^a^								
RM 2.50 and less or ≤ USD 0.80 per day (*n* = 175)	0.67	0.93	0.44	0.73	0.33	0.60	0.44	0.73
	(0.56–0.89)	(0.80–1.00)	(0.22–0.67)	(0.53–0.87)	(0.11–0.44)	(0.40–0.73)	(0.22–0.56)	(0.60–0.87)
More than RM 2.50 or > USD 0.80 per day (*n* = 227)	0.78	0.93	0.33	0.73	0.33	0.60	0.44	0.73
	(0.56–0.89)	(0.87–1.00)	(0.22–0.56)	(0.53–0.87)	(0.22–0.44)	(0.40–0.80)	(0.22–0.56)	(0.60–0.87)
Internet Surfing Time (ST)^a^								
ST ≤30 min (*n* = 220)	0.67	0.93	0.33	0.73	0.22	0.60	0.44	0.73
	(0.44–0.89)	(0.80–1.00)	(0.22–0.56)	(0.53–0.87)	(0.11–0.44)	(0.40–0.78)	(0.22–0.56)	(0.60–0.87)
ST >30 min (*n* = 182)	0.78	0.93	0.44	0.73	0.33	0.60	0.44	0.73
	(0.56–1.00)	(0.87–1.00)	(0.22–0.56)	(0.53–0.87)	(0.22–0.44)	(0.40–0.80)	(0.22–0.56)	(0.53–0.87)
Household Income								
Low [≤RM 2300 or ≤ USD 720 per month] (*n* = 190)	0.78	0.93	0.44	0.73	0.33	0.60	0.44	0.73
	(0.56–0.89)	(0.80–1.00)	(0.22–0.67)	(0.53–0.87)	(0.11–0.47)	(0.40–0.80)	(0.22–0.56)	(0.60–0.87)
Medium and high [>RM2300 or > USD 720 per month] (*n* = 212)	0.78	0.93	0.33	0.73	0.33	0.60	0.39	0.67
	(0.56–0.89)	(0.87–1.00)	(0.22–0.56)	(0.53–0.87)	(0.11–0.44)	(0.42–0.80)	(0.22–0.56)	(0.60–0.87)

M = median, IQR = interquartile range, core = core foods, non-core = non-core foods

Note: Core foods are defined as low-calorie, nutrient-dense food products, while non-core foods are foods high in fat, refined sugars, and salt (HFSS) [33]

^a^Categorisation for influencing factors was based on median of subjects

*Significance for core to non-core food in each induction factor comparison were recorded as *p* < 0.001

### Non-core food induction factors *vs* influencing factors

**Fig. 3:**
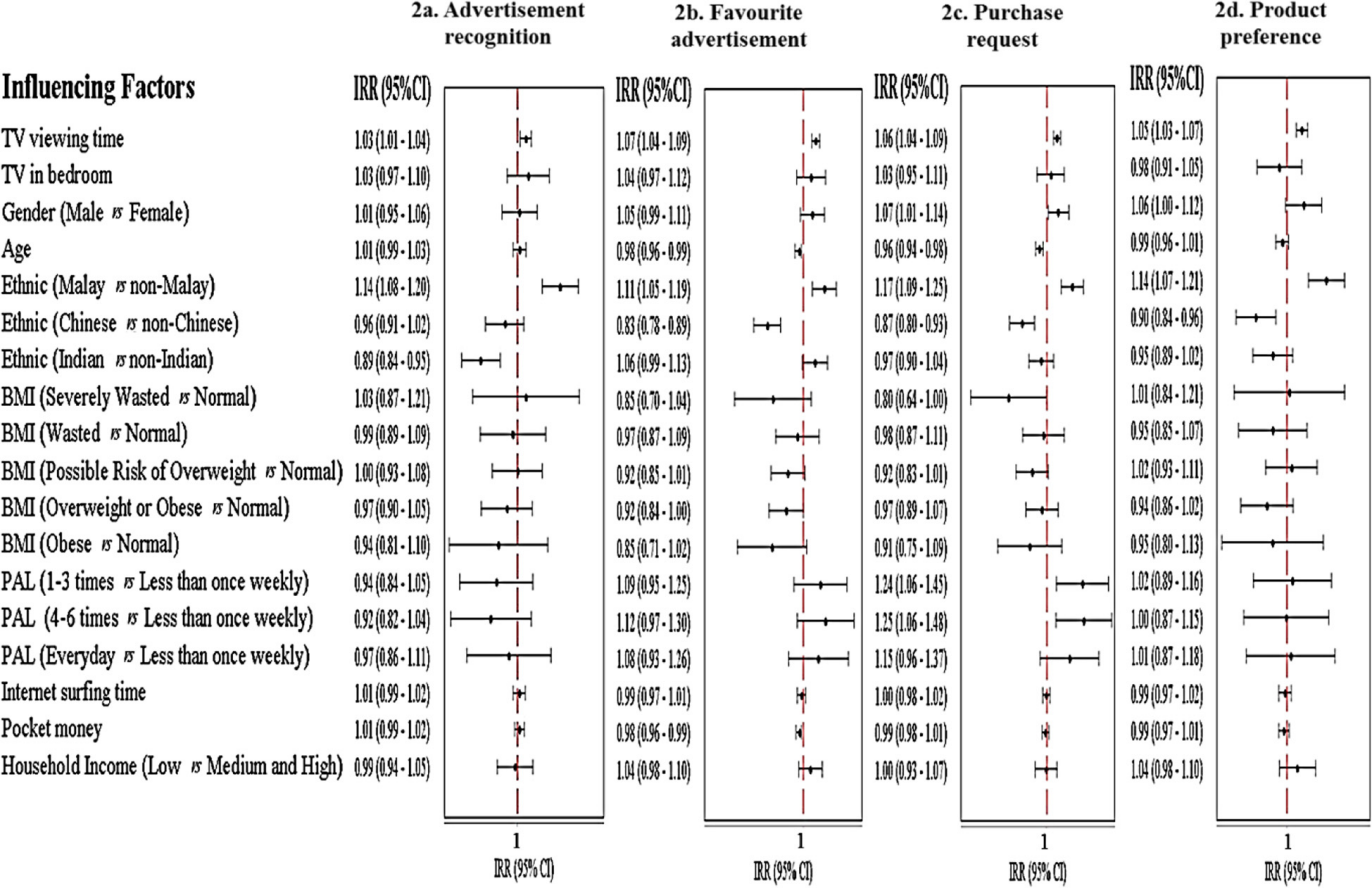
**a**-**d** Weighted unadjusted incidence rate ratio (IRR) for induction factors related to non-core TVFA targeting children. Incidence rate ratio of scores for (**a**) advertisement recognition, (**b**) favourite advertisement, (**c**) purchase request, and (**d**) product preference related to influencing factors such as TV viewing time, TV in bedroom, gender (Male *vs* Females), age, ethnic (Malay *vs* Non-Malay; Chinese *vs* Non-Chinese; Indian *vs* Non-Indian), body mass index of children [BMI] (Severely wasted or Wasted or Possible risk of overweight or Overweight/ Obese or Obese *vs* normal), physical activity level [PAL] (1–3 times or 4–6 times or Everyday *vs* Less than once weekly), daily internet surfing time, daily pocket money and monthly household income (Low: ≤RM2300 *vs* Medium and high: >RM2300). Note: A vertical line represents incidence rate ratio of 1. If the value of 1 falls within 95 % confidence interval, there is no significant association between tested induction factors and influencing factors (*p* > 0.05).

Results from univariate Poisson regression analyses for each induction factor are presented in the form of Forest plots in Fig. 3a-d. TV viewing time (IRR: 1.03; 95 % CI: 1.01–1.04) and ethnicity (Malay *vs* non-Malay, IRR: 1.14; 95 % CI: 1.08–1.20) were significantly associated with advertisement recognition as shown in Fig. 3a. Favourite advertisement (Fig. 3b) was significantly associated with TV viewing time (IRR: 1.07; 95 % CI: 1.04–1.09), age (IRR: 0.98; 95 % CI: 0.96–0.99), ethnicity (Malay *vs* non-Malay, IRR: 1.11; 95 % CI: 1.05–1.19), and daily pocket money (IRR: 0.98; 95 % CI: 0.96–0.99). In the context of purchase request, TV viewing time (IRR: 1.06; 95 % CI: 1.04–1.09), gender (IRR: 1.07; 95 % CI: 1.01–1.14), age (IRR: 0.96; 95 % CI: 0.94–0.98), ethnicity (Malay *vs* non-Malay, IRR: 1.17; 95 % CI: 1.09–1.25), and physical activity level (1–3 times *vs* less than once weekly, IRR: 1.24; 95 % CI: 1.06–1.45 and 4–6 times *vs* less than once weekly, IRR: 1.25; 95 % CI: 1.06–1.48) were significantly associated with purchase request (Fig. 3c). Additionally, product preference (Fig. 3d) was significantly associated with children’s TV viewing time (IRR: 1.05; 95 % CI: 1.03–1.07) and ethnicity (Malay *vs* non-Malay, IRR: 1.41; 95 % CI: 1.07–1.21).

### The effect of TV viewing time on induction factors for non-core foods

In multivariate analysis, when corrected for other variables, TV viewing time was still significant (*p* < 0.001) with favourite advertisement (IRR_final adj_: 1.06; 95 % CI: 1.04–1.08), purchase request (IRR_final adj_: 1.06; 95%CI: 1.04–1.08) and product preference (IRR_final adj_: 1.04; 95 % CI: 1.02–1.07), but not advertisement recognition (IRR_final adj_: 1.02; 95%CI: 1.00–1.04) (Table 4).

**Table 4:** Effect of TV viewing time on induction factors for non-core food advertising after correcting for all influencing factors

Induction factors	IRR_final adj_ (95 % CI)
Advertisement Recognition	1.02 (1.00–1.04)
Favourite Advertisement	1.06 (1.04–1.08)^*^
Purchase Request	1.06 (1.04–1.08)^*^
Product Preference	1.04 (1.02–1.07)^*^

Dependent variable was score of each induction factors. Independent variable was TV viewing time and adjusted for other influencing factors such as (i) ethnicity, (ii) BMI status of child, (iii) age of child, (iv) gender, (v) TV set in bedroom, (vi) physical activity of child daily, (vii) daily pocket money of child, (viii) daily internet surfing time of child, and (ix) monthly household income

*Poisson regression model with the significance level at *p* < 0.05

### Influence of ethnicity on induction factors for non-core foods

In the analyses by ethnic groups, TV viewing time was not associated with non-core food recognition across all ethnic groups (Table 5). Generally, for every additional hour of TV viewing amongst Malay children, the incidence rates for them to find non-core food advertisements to be attractive (IRR_adj:_ 1.05; 95 % CI: 1.01–1.08), purchase request on these products (IRR_adj_: 1.06, 1.02–1.10) and prefer these types of foods (IRR_adj:_ 1.04; 95 % CI 1.01–1.08) were significantly higher. Similarly, the incidence rates for every additional hour of TV viewing by Indian children were significantly increased for favourite advertisement (IRR_adj_: 1.06; 95 % CI: 1.02–1.10), purchase request (IRR_adj_: 1.05; 95%CI: 1.01–1.09) and product preference (IRR_adj_: 1.05; 95 % CI: 1.01–1.09). However, TV viewing time for Chinese children was not significantly associated (*p* > 0.05) with all induction factors except favourite advertisements (IRR_adj_: 1.06; 95 % CI: 1.01-1.10).

**Table 5:** Effect of TV viewing time on induction factors for non-core food advertisements as per ethnicity

Ethnic Group	Advertisement Recognition	Favourite Advertisement	Purchase Request	Product Preference
	IRR_adj_ (95 % CI)	IRR_adj_ (95 % CI)	IRR_adj_ (95 % CI)	IRR_adj_ (95 % CI)
Malay	1.01 (0.98–1.05)	1.05 (1.01–1.08)*	1.06 (1.02–1.10)*	1.04 (1.01–1.08)*
Chinese	1.02 (0.98–1.06)	1.06 (1.01–1.10)*	1.07 (0.90–1.25)^a^	1.04 (0.99–1.09)
Indian	1.02 (0.98–1.05)	1.06 (1.02–1.10)*	1.05 (1.01–1.09)*	1.05 (1.01–1.09)*

Dependent variable was score of each induction factors. Independent variable was TV viewing time and adjusted for other influencing factors such as (i) BMI status of child, (ii) age of child, (iii) gender, (iv) TV set in bedroom, (v) daily physical activity of child, (vi) daily pocket money of child, (vii) daily internet surfing time of child, and (viii) monthly household income

^a^Based on negative binomial procedure

*Poisson regression model with the significance level at *p* < 0.05

### Why would children be attracted to TVFA?

**Fig. 4:**
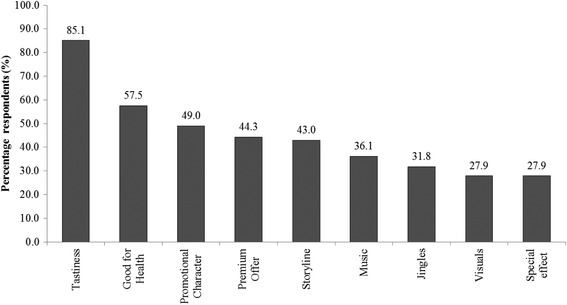
Attractive reasons of food advertisements targeting children. Cited reasons among children to be attracted towards favourite advertisements (*n* = 402)

Almost four in five children responded that ‘tastiness of advertised food product’ was the topmost attraction to like a food advertisement (Fig. 4). More than half of children (*n* = 231, 57.5 %) said they would prefer advertised foods if the advertisements were labelled as good for health. Persuasive techniques used by food advertisers in TV commercials such as product endorsements with promotional characters (49.0 %), premiums offers such as free gifts (44.3 %) and using a storyline (43.0 %) were reported to gain children’s attention. However, just one in four children reported an interest in TVFA if they carried elements of colourful visuals and special effects.

## Discussion

Our study in urban Kuala Lumpur confirmed a reproducible pattern of children’s TV viewing time compared to a previous Malaysian study conducted in Sabah state [8]. During weekends, approximately 44 % children watched more than 3 h per day of TV compared to fewer hours during weekdays [8]. In our study, self-reported TV viewing time indicated 191 children (47.5 %) spent three hours or more watching TV daily (Table 3). However, this figure of 47.5 % reported for this Malaysian study was comparatively higher to Thailand and Nigeria where parental reports indicated 28 % and 36 % of children respectively, spent ≥3 h daily viewing TV [41]. A longer TV viewing time suggests a greater exposure to TVFA could influence preferences for, and possibly increases frequency of consumption of, unhealthy advertised foods such as soft drinks, snacks and fast foods among children [42–44].

Findings in this study revealed that Malaysian school children were more attracted to unhealthy TV advertisements than healthier core food advertisements. For every additional hour of TV viewing, the probability for children to like non-core food advertisements as their favourite TV advertisements increases by 1.05 to 1.06 times, irrespective of their ethnic groups. The favourite advertisements of the studied children were sugar-sweetened beverage, fast food, ice-confectionery, high sugar and/or low fibre breakfast cereal and extruded snack (Brand 2). We observed these products shared similar characteristics to obtain market share, such as being tasty (enhanced by high refined sugar, fat and salt), endorsed by promotional characters that were branded and offered free gift. All these techniques would draw children’s attention when exposed to TVFA. In particular toy premiums or giveaways were the most common techniques used by fast food restaurants on TV to target children [45].

Generally, most children in this study recognised 13/15 advertised food products from the non-core foods category shown in the food album. Sugar sweetened beverages, high sugar and/ or low fibre breakfast cereal, fast food and ice-confectionery were non-core foods commonly recognised. Interestingly, we observed a parallel scenario between the types of food products recognised by children in this study with exposure rates for non-core food advertisements on Malaysian TV channels [34]. However, our observations showed that children’s recognition of non-core foods was partially mediated by the TV viewing duration (IRR: 1.03; 95 % CI 1.01 to 1.04) in a univariate analysis. Rather, in Malaysia, ethnicity plays a major role in children's advertisements recognition as a local content analysis of three major ethnic channels highlighted there was a significant difference in food advertising exposure between ethnic-specific channels [32]. We also noted that the probability for Malay children to recognise non-core food advertisements was 1.14 times higher compared to the other ethnic groups. Perhaps, this phenomenon could be explained by a local content analysis showing high rates of non-core food exposure observed in Malay-centric children’s popular TV channels, in particular during school holidays [32]. Further analysis of the association between TV viewing time as per ethnicity and non-core food recognition after adjustment for influencing factors was not significant (*p* < 0.05). This could be due to high non-core food recognition score for all ethnic groups (median score as 0.93) and hence TV viewing time became irrelevant.

Age is an influencing factor to determine effectiveness of TVFA. Our univariate analyses showed that the probability of children to perceive advertised non-core foods as favourite advertisements (IRR: 0.98; 95 % CI: 0.96-0.99) and trigger purchase request for these foods (IRR: 0.96; 95 % CI: 0.94-0.98) reduced as they become older. Perhaps, this observation could be explained as younger children usually recall more peripheral information and display false beliefs about foods using persuasive marketing techniques rather than the products nutritional attributes [46].

In this study, every additional hour of TV viewing increased the incidence rate of purchase request amongst Malay and Indian children for advertised non-core foods by 1.05 to 1.06 times, after correcting for other influencing factors. In our previous study, Indian popular TV channels in Malaysia screened very little TVFA compared to other ethnic centric channels [32]. It is therefore probable that Indian children were less familiar with food advertising and hence are inexperienced in navigating food commercial messages. As a result, Indian children might become vulnerable to induction into purchase requesting behaviour with increased TV viewing time as observed in this study. Ghimire and Rao [47] indicated that Indian children were more likely to purchase advertised products if their favourite models or actors were in the TV advertising. In contrast, children who did not initiate purchase requests after long TV viewing time and exposure to TVFA, could be due to self-repression of this desire. Mehta et al. [48] hypothesize repression may be due to parental ignoring of the first request which develops into conditioning behaviour to anticipated parental refusal as the child grows older.

Notably, for every additional hour of TV viewing time, the probability of Malay and Indian children to prefer advertised non-core food products increases by 1.04 and 1.05 times, respectively. In a prospective cohort study in Denmark, Hare-Bruun et al. [49] found that TV viewing time was significantly associated with unhealthy food preferences and food habits, which might be a result of TV commercials. Fundamentally, the model of persuasion explains that changes in children’s attitudes and behaviour caused by food advertising is attributed to the combination of attractiveness and credibility of sources, incentives and repetition of messages [50]. Therefore, credible celebrity endorsement in food advertisements might be mistaken by children as ‘super foods’. In the social learning theory proposed by Bandura [51], children would imitate the behaviour of these characters to consume foods endorsed by them and further establish food preference at an early age.

In this study, effect of watching TV time among Chinese children did not show any association with induction factors except favourite advertisements. We observed that Chinese vernacular schools in urban Malaysia place great emphasis on school grade achievement, hence students were often given greater school workload. This is evidenced by TV viewing time amongst Chinese children being the lowest compared to Malay and Indian children (p < 0.05). Hence, the focus on academic achievement in Chinese children attending vernacular schools could perhaps act as a mitigating mechanism to reduce exposure to TVFA in Malaysia.

In summary, our findings indicate every additional hour of TV viewing would affect children’s attitudes towards non-core TVFA. Thus, these advertisements become incrementally appealing causing these food products more likely to be requested and preferred. Of concern, children who repress their purchase request might translate induction effects of TVFA into positive attitudes particularly for non-core food products and this could become a future health risk for children [52]. Contemporary social cognitive theory explains food advertising might increase food consumption without hunger, advertising awareness or mood factors [53]. If pre-established attitudes persist into adulthood, this could be the biggest challenge for young people to reverse their positive attitudes towards unhealthy foods. Defining proper age range to protect children from TVFA in policy development has been called for by public health professionals [21, 23]. This study will therefore contribute a better understanding on the impact of TVFA on children and add insights into future policy development.

This research serves as a single, self-reported and cross-sectional study to evaluate impacts of TVFA on four induction factors. Further longitudinal studies would better elucidate information about the long term impacts of TVFA on children. An acknowledged limitation was we did not assess actual caloric intake and nutrients of these advertised products to translate into a measure of actual consumption by the children. Apart from this, children recruited were from a metropolitan area and these results need to be interpreted cautiously when generalised to populations covering rural areas. The strength of this study was that it included three major ethnic groups and trained interviewers were able to converse in the language preferred by children (such as Mandarin, Tamil, Malay or English).

## Conclusions

Our findings indicate that food industries in Malaysia have successfully manipulated the mind of children by using attractive TV commercials, promoting purchase requests and instilling early childhood preference for non-core foods. We observed a difference in  media consumption pattern and advertisement attitudes between ethnic groups, which should be critically considered in policy development. The observation that early food preferences might persist into young adulthood happens when the mind of young school children essentially switches into becoming lifelong consumers. Any efforts to initiate prevention in early childhood would be viewed as critical to protect the children from any misleading exaggerated claims from food advertisements, especially unhealthy TVFA.

## Additional file

Additional file 1:
**Questionnaire survey.** (DOCX 327 kb)

## Abbreviations

ANOVA: Analysis of variance
BMI: Body mass index
CI: Confidence interval
HFSS: Foods high in fat refined sugars and salt
IQR: Interquartile range
IRR: Incidence rate ratio (unadjusted)
IRR_adj_: Adjusted incidence rate ratio
IRR_final adj_: Final adjusted incidence rate ratio
M: Median
NCDs: Non-communicable diseases
PAL: Physical activity level
ST: Internet surfing time
TV: Television
TVFA: Television food advertising
UK: United Kingdom
