# Supplementary material for: Reading the mind of children in response to food advertising: a cross-sectional study of Malaysian schoolchildren’s attitudes towards food and beverages advertising on television
Source: BMC Public Health. 2015 Oct 12;15:1047. doi: 10.1186/s12889-015-2392-z (PMC4603941; doi:10.1186/s12889-015-2392-z)
Supplement: Additional file 1: — Questionnaire survey. (DOCX 327 kb) [file 12889_2015_2392_MOESM1_ESM.docx]

**QUESTIONNAIRE SURVEY**


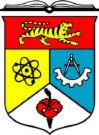


**DIETETICS PROGRAM**

**SCHOOL OF HEALTHCARE SCIENCES**

**FACULTYOF HEALTH SCIENCES**

**UNIVERSITI KEBANGSAAN MALAYSIA**

**KUALA LUMPUR**

**STUDY ON FOOD ADVERTISING ON**

**MALAYSIAN TELEVISION**

**TARGETING CHILDREN**

**Attention to parent/ guardian….**

**Proceed to answer this questionnaire if you fulfill these criteria:**

1. **Full-time/ partially parenting (i.e. working but takes care child at night) and**
2. **Child (aged 7-12) watches TV at least half an hour per week.**

*If you DO NOT fulfill the above 2 criteria, your participation is not required.*

**Parent’s Name : _________________________________________________**

**Child’s Name : _________________________________________________**

**Date of Birth (Child) : _____________________**

**Child’s School Name: _____________________ Class: _____________________**

**NOTE THE RESEARCHER:**

The child referred in the questionnaire only applies to the child belonging to the parent/ guardian cited in Part I. Only one child per consenting parent/ guardian can be interviewed. This means his/ her siblings will not be sampled in this study.

**QUESTIONNAIRE SURVEY**

**PART I: SOCIODEMOGRAPHIC INFORMATION OF PARENT/GUARDIAN (FILLED IN BY CAREGIVER)**

| **Biodata Information** | | | | | | | | | |
| --- | --- | --- | --- | --- | --- | --- | --- | --- | --- |
| 1. | Name : | | | | Relationship with child:  Mother Father Guardian | | | | |
| 2. | Age (years): | | | | | | | | |
| 3. | IC (New): | | | | | | | | |
| 4. | H/P (House): H/P (mobile): | | | | | | | | |
| 5. | Marital Status:  Single Married Divorced Widow/ widower | | | | | | | | |
| 6. | Education Level:  No Schooling Primary School Secondary School  College/ University  Cert:  Diploma Degree Master PhD | | | | | | | | |
| 7. | Occupation :___________________ | | | Retired/ Unemployed Self-employed  Government Sector Private Sector | | | | | |
| 8. | Household Income (*including spouse if any*):  RM 2,300 and below  More than RM 2,300 – RM 5,599  RM 5,600 and above | | | | | |  | | |
| 9. | How much **POCKET MONEY** do you give **YOUR CHILD** per day? | | | | | Total RM : __________ per day | | | |
| 10. | No. | Name of Siblings | Age | | | School’s name | | Class | Same school as participant |
|  | 1. |  |  | | |  | |  |  |
|  | 2. |  |  | | |  | |  |  |
|  | 3. |  |  | | |  | |  |  |

**PART II: QUESTIONNAIRE SURVEY ON CHILDREN’s TV HABITS**

**(INTERVIEWER ADMINISTERED)**

The child referred in the questionnaire only applies to the child belonging to the parent/ guardian cited in Part I. Only one child per consenting parent/ guardian can be interviewed. This means his/ her siblings will not be sampled in this study.

**Background of the child**

Please **TICK (√)** or **fill in the blank** with your answer.

1. Name :________________________________________________________
2. Age :_______ year
3. Gender : Male Female
4. Race : Malay Chinese Indian Other:____________
5. School Name : ________________________ Class: _______________
6. How many TV(s) do you have in your household? ______________

| Yes | No |
| --- | --- |

1. Do you have a TV in your bedroom?

| Yes | No |
| --- | --- |

1. Do you have internet access at home?

(e.g. smartphone/ broadband/ streamyx)

**If YES:**

How much time on average do you access the internet per day? _____ hour (s) _____ minutes per day?

| Less than once a week | 1-3 times a week | 4-6 times a week | every day | Don’t know (Don’t read) |
| --- | --- | --- | --- | --- |

9. How often do you engage in physical activity or exercise (include “*Physical Education*”) in the past one week?

Types of physical activity: __________/____________/_____________/____________

If you engage in physical activity, how much time on average do you perform it per day?

____ hour (s) ____ minutes per day

10. Generally, how much time* will you spend watching TV on a weekday and weekend? *draw a line to indicate it.

**[Interviewer asks- How many hour(s) on average does the child spend watching TV daily? ___ hour (s) ___ minutes per day]**

**WEEKEND**


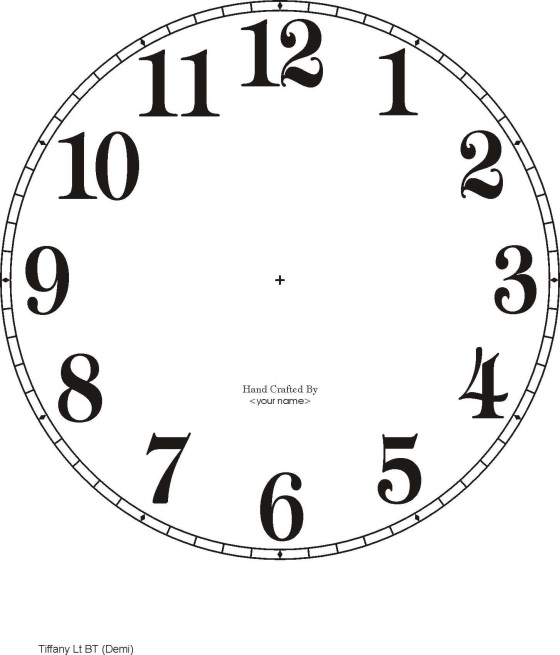
**
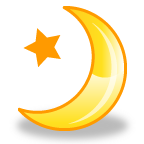

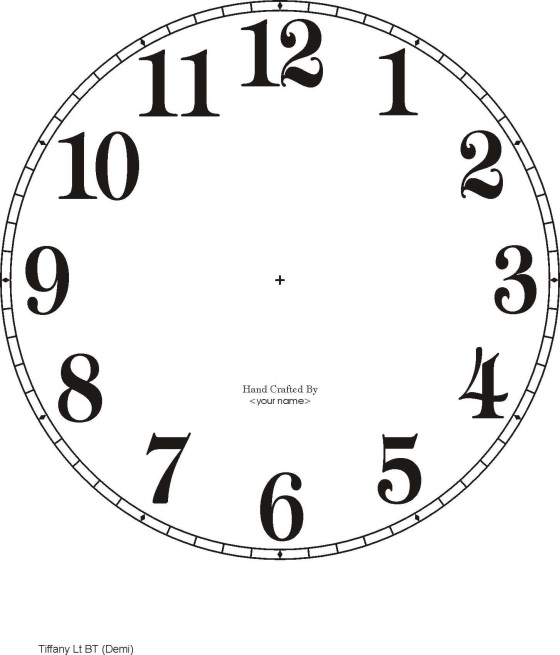

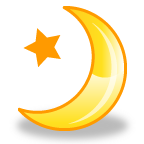
**
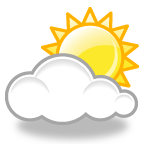

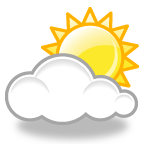

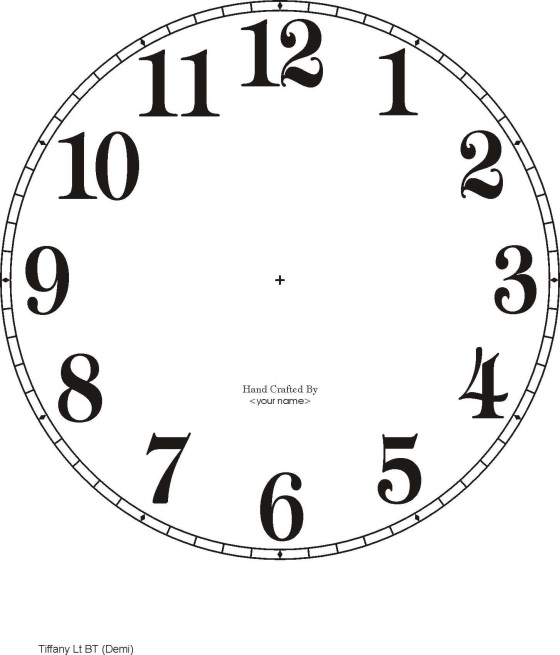

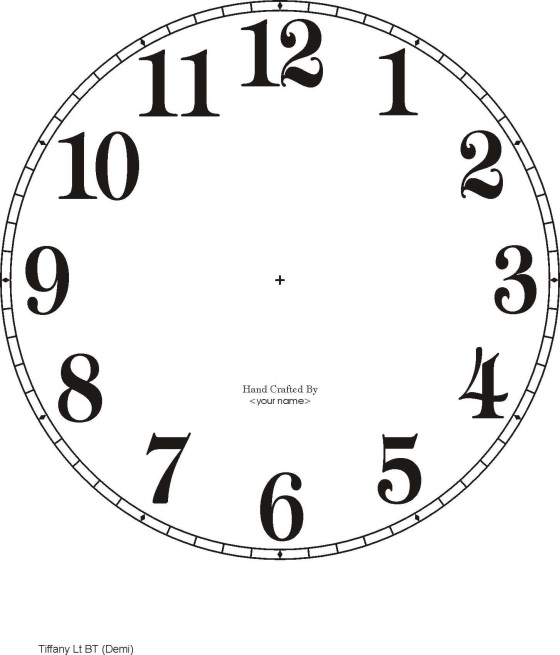

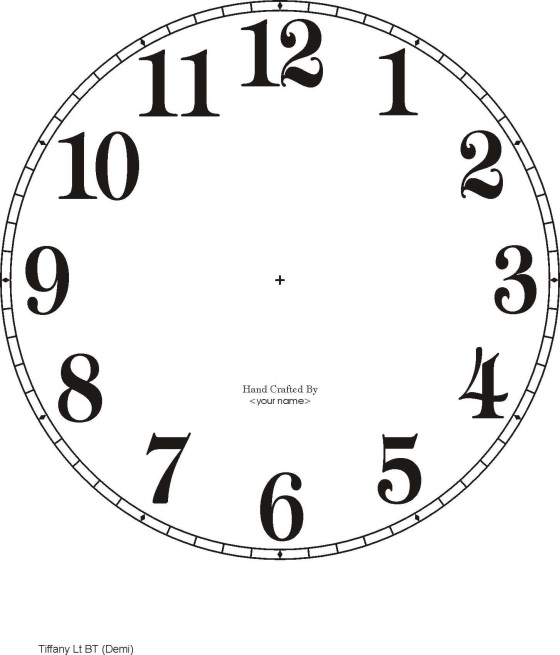

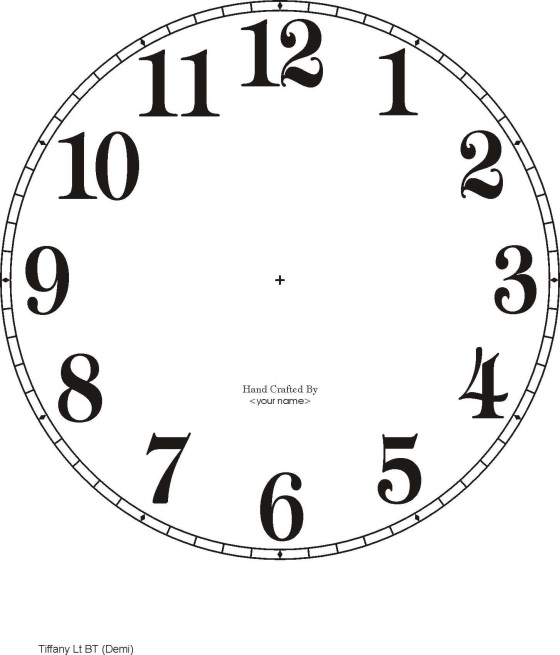


**Example: watch TV from 8pm till 10 pm**

**WEEKDAY**

**p.m. (weekday)**

**p.m. (weekend)**

**a.m. (weekend)**

**a.m. (weekday)**

**Food Preference of the Child**

**Food Album Questionnaire**

Please indicate your answer as: **√: Yes; X: No**

| Food Product (FP) | Have you seen this TV advertisement before? | Do you like the advertisement? | 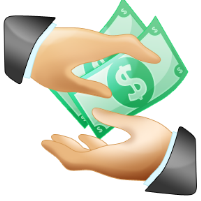Will you ask your parent to buy it? | Do you like to eat/ drink this food product? |
| --- | --- | --- | --- | --- |
| FP1 |  |  |  |  |
| FP2 |  |  |  |  |
| FP3 |  |  |  |  |
| FP4 |  |  |  |  |
| FP5 |  |  |  |  |
| FP6 |  |  |  |  |
| FP7 |  |  |  |  |
| FP8 |  |  |  |  |
| FP9 |  |  |  |  |
| FP10 |  |  |  |  |
| FP11 |  |  |  |  |
| FP12 |  |  |  |  |
| FP13 |  |  |  |  |
| FP14 |  |  |  |  |
| FP15 |  |  |  |  |
| FP16 |  |  |  |  |
| FP17 |  |  |  |  |
| FP18 |  |  |  |  |
| FP19 |  |  |  |  |
| FP20 |  |  |  |  |
| FP21 |  |  |  |  |
| FP22 |  |  |  |  |
| FP23 |  |  |  |  |
| FP24 |  |  |  |  |

1. Why is/ are the listed advertised food/ beverages product(s) attractive to you?

| Storyline | Visuals | Special effect | Music (tunes/ songs) |
| --- | --- | --- | --- |
| Jingles | Tasty | Prize/ giveaways/ competition | Promotion characters (cartoon/ celebrities) |
| Good for health | Others:______________________ | |  |

**Anthropometry Measurement of Child**

| **Parameter** | **R1** | **R2** | **Average** |
| --- | --- | --- | --- |
| 1. **Weight (kg)** |  |  |  |
| 1. **Height (m)** |  |  |  |
| 1. **BMI (kgm^-2^)** |  | | |

~ The End. Thank You ~
